# Tensile Strength of Poly(lactic acid)/Bleached Short Hemp Fiber Fully Green Composites as Replacement for Polypropylene/Glass Fiber

**DOI:** 10.3390/polym15010146

**Published:** 2022-12-28

**Authors:** Roberto J. Aguado, Francesc X. Espinach, Fernando Julián, Quim Tarrés, Marc Delgado-Aguilar, Pere Mutjé

**Affiliations:** LEPAMAP-PRODIS Research Group, University of Girona, C. Maria Aurèlia Capmany, n° 61, 17003 Girona, Spain

**Keywords:** biocomposites, cellulose, dispersion, fiber–matrix interface, micromechanics, natural fibers, poly(lactic acid), short fiber reinforcement, tensile strength

## Abstract

The compatibility between poly(lactic acid) (PLA) and natural fibers to develop bio-sourced, recyclable, and biodegradable composites remains a commonplace issue. This work highlights that, at least in the case of hemp, pulping and bleaching towards delignified short fibers attained remarkable improvements over untreated hemp strands. This approach differs from usual proposals of chemically modifying hydroxyl groups. Soda-bleached hemp fibers (SBHFs) granted a relatively large bonding surface area and a satisfactory quality of the interphase, even in the absence of any dispersant or compatibilizer. To attain satisfactory dispersion, the matrix and the fibers were subjected to kinetic mixing and to a moderately intensified extrusion process. Then, dog-bone specimens were prepared by injection molding. Up to a fiber content of 30 wt.%, the tensile strength increased linearly with the volume fraction of the dispersed phase. It reached a maximum value of 77.8 MPa, signifying a relative enhancement of about 52%. In comparison, the tensile strength for PLA/hemp strands was 55.7 MPa. Thence, based on the modified rule of mixtures and the Kelly & Tyson modified equation, we analyzed this performance at the level of the constituent materials. The interfacial shear strength (over 28 MPa) and other micromechanical parameters were computed. Overall, this biocomposite was found to outperform a polypropylene/sized glass fiber composite (without coupling agent) in terms of tensile strength, while fulfilling the principles of green chemistry.

## 1. Introduction

The UN Environmental Assembly of February and March of 2022 assigned top priority to a globally binding agreement on plastic pollution [1]. Far from being a mere symbolic gesture, the legislative pressure on manufacturing industries is increasing, including the ongoing European Strategy for Plastics in a Circular Economy [2]. In its second chapter, the referred communication highlights the opportunities brought by “plastics with biodegradable properties”. One of the most popular choices for such an endeavor is poly(lactic acid) (PLA), which, besides being biodegradable (at least at high temperature), can be recycled both chemically and mechanically [3]. Nevertheless, its usage, like that of any other biopolymer, has severe limitations when it comes to challenging the so-called *big four*—polyethylene, polypropylene (PP), polystyrene, and poly(vinyl chloride) [4]. A promising strategy to enhance its properties without hampering its biodegradability is its reinforcement with wood or plant fibers towards fully bio-sourced composites [5,6,7,8].

While the global market size of biocomposites is growing [9], it still entrails conventional composite materials with oil-sourced matrices and energy-intensive reinforcement fibers, namely PP/glass fiber (GF) [10,11]. Some of the most convincing natural fibers, such as hemp, are easy to grow and harvest, and they are environmentally friendlier than GF [12], but fall shorter in terms of performance. Even PLA has been reinforced with GF towards an 84% improvement in its tensile strength [13]. Limitations of hemp fibers and other natural fibers include lower intrinsic tensile strength values, high surface polarity, and ease of water sorption (which is detrimental to molten PLA [6]). The possibility of overcoming them justifies further investigation.

Previous works have suggested different approaches to improve the compatibility with wood fibers. For instance, performing chemical modifications on natural fibers, such as silane coupling or esterification with acetic anhydride, has been proven to enhance the dispersion of the fibers in the matrix and to avoid self-bonding [14]. In a more simple approach, the removal of hemicelluloses and pectin from hemp, while keeping the lignin content at 1.4 wt.%, sufficed to obtain remarkable enhancements in performance [15]. For similar purposes, we have reinforced PLA with a bleached pulp from eucalyptus wood [16], using diethylene glycol dimethyl ether as a dispersant. Nonetheless, the use of oil-based additional components that are, in addition, lost by evaporation during processing, contradicts at least two principles of green chemistry, namely atom economy and the choice of renewable materials [17].

Despite their usefulness, derivatization processes, the use of compatibilizers and the presence of excess reagents increase the environmental impact of the manufacturing process. In addition, while the environmental performance of alkaline pulping and bleaching should keep improving, they are feasible and traditional processes that, over the course of time, have attained high material efficiency [18].

Hemp strands, both untreated and after undergoing different chemical processes, have already been incorporated into PLA matrices [14,19,20]. Song et al.’s [19] biocomposites, comprising PLA and degummed hemp strands, reached tensile strength improvements of up to 39% over the initial bioplastic. We hypothesize that shorter, discontinuously dispersed fibers, with lower contents of lignin and hemicellulose, would attain greater interfacial shear strength after extensive blending processes. This hypothesis is hereby tested with soda-bleached hemp fibers (SBHFs). It is expected that SBHFs offer more surface area for intermolecular interactions [21].

All considered, this work tests the tensile strength of PLA/SBHF composites, differing from the most usual approaches in that: (i) they contain no compatibilizer or dispersant; (ii) etherifications, esterifications, and silane coupling reactions are avoided. To grant good dispersion of the fibers in the matrix, blending implied moderately intensifying the extrusion process before injection molding. First, key differences between hemp strands and short fibers obtained thereof are exposed in terms of their dimensions and their basic chemical composition. Then, the results are analyzed to elucidate the quality of the interaction and the micromechanical parameters. Once the interfacial shear strength is computed, fiber-matrix intermolecular interactions are discussed. Finally, these fully green biocomposites are found to match PP/GF composites in terms of their tensile strength.

## 2. Materials and Methods

### 2.1. Materials

The commercial PLA referred to in this article is Ingeo ™ Biopolymer 3251D by NatureWorks (Plymouth, MN, USA). Its density is 1.24 g cm^−3^, its melting point is approximately 160–170 °C, and its melt flow rate (190 °C, 2.16 kg) is 35 g/10 min.

Hemp strands were kindly provided by Agrofibra S.L. (Puigreig, Catalonia, Spain). A soda-bleached pulp from hemp (elemental chlorine and total chlorine free), ISO brightness 89.5%, was provided by Celesa (Tortosa, Catalonia, Spain). Before blending, dry pulp boards were fractionated by passing through a paper shredder.

All the reagents employed to characterize SBHFs and untreated hemp strands (UHSs) were purchased from Scharlab S.L. (Sentmenat, Catalonia, Spain) and used as-is. Poly(vinyl sulfate) and methylglycol chitosan (MGCh) were acquired from Wako Chemicals, GmbH (Neuss, North Rhine-Westphalia, Germany).

### 2.2. Characterization of Constituents

We carried out a basic chemical characterization of SBHFs and UHSs by following common TAPPI standards [22], namely: T 264 cm-07 for sample preparation, T 204 cm-17 for extractives, T 211 sp-11 for the ash content, T 249 cm-21 for hemicellulose, T 429 cm-10 for cellulose, and T 222 om-15 and UM 250 for lignin. The average dimensions of fibers, along with the percentage of fines, were computed using a MorFi Compact analyzer from Techpap (Gières, Isère, France) and its software MorFi v9.2. The crystallinity index was estimated from X-ray diffraction patterns and by the Segal method, as described elsewhere [23].

The surface polarity of SBHFs and PLA was assessed by a colloidal back titration. For that, both pulp fibers and previously frozen PLA pellets were screened in the same way (200 mesh). In short, excess MGCh was added to an aqueous suspension of fibers or PLA, the mixture was briefly stirred (45 s), and then it was centrifuged at 2000× *g* for 15 min. The supernatant was titrated with potassium poly(vinyl sulfate) as a titrating agent and toluidine blue O as an indicator.

### 2.3. Processing

Biocomposites were produced by combining the PLA matrix with 10–30 wt.% of reinforcement fibers (SBHFs). They were passed through a Gelimat ™ kinetic mixer, model G5S, from Dusatec (Ramsey, NJ, USA). The rotor speed was 2500 rpm, the processing time was 3 min, and the discharge temperature was 200 °C. To obtain as much fiber dispersion as possible, these blends were extruded twice in a single-screw machine from Eurotecno (Sabadell, Catalonia, Spain), model 3035 D. Screw speed was 40 rpm and the temperature profile ranged from 180 °C to 205 °C. Then, we granulated the extrudate by grinding it in a hammer mill and stored it at 80 °C for 24 h.

**Figure 1:**
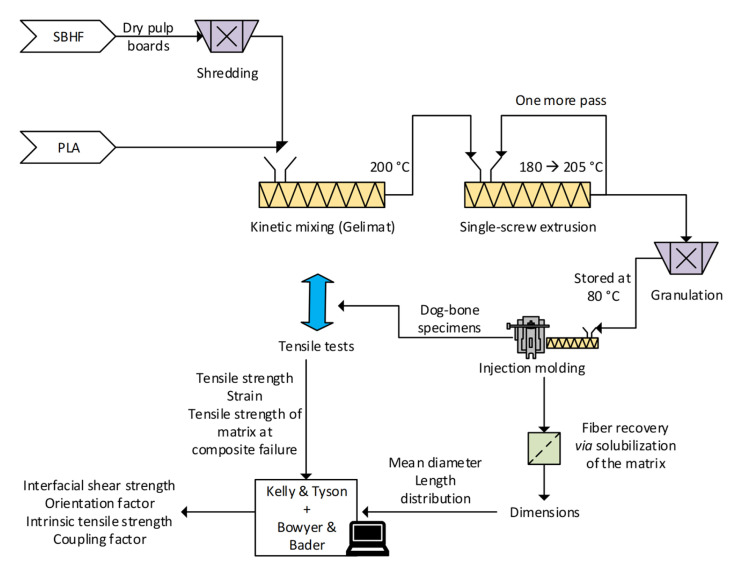
Depiction of the experimental procedure, schematizing preparation, testing and micromechanical analysis.

The procedure is schematized in Figure 1, which also refers to the biobased sourcing of both fibers and matrix.

### 2.4. Injection and Characterization of Composites

Specimens for tensile tests were produced using an injection molding machine from Arburg (Lossburg, Baden-Wurtemberg, Germany), model 220 M 350-90U. The processing temperature increased from 170 °C (hopper side) to 210 °C (nozzle). The injection pressure was set at 50 MPa for PLA-only samples, 60 MPa for a fiber load of 10 wt.%, 70 MPa for 20 wt.%, and 80 MPa for 30 wt.% SBHFs. Specimens (dog-bone type I, narrow section 57 mm × 13 mm), were conditioned under standard conditions of temperature (23 °C) and relative humidity (50%), according to ASTM D618 [24].

Up to ten dog-bone samples were submitted to tensile tests through a Universal Testing Machine from Instron (Barcelona, Catalonia, Spain), model 1122, including a 5 kN load cell and an extensometer. The test speed was 2 mm/min, following the ASTM standard D3039 [25].

Since mechanical and heat stresses are known to impart changes in fiber morphology, fibers were extracted from each composite sample. Briefly, dichloromethane was used for the selective dissolution of the matrix, and the residual PLA in the remaining solid was removed using Soxhlet extraction with decalin [26]. The dimensions of recovered fibers were computed employing the MorFi Compact analyzer as described above.

Following tensile tests, micrographs were obtained from the fracture section of specimens by means of a ZEISS DSM 960A (ZEISS Iberia, Madrid, Spain) scanning electron microscope (SEM), coupled to a secondary electron detector. For that, samples were subjected to carbon coating and the voltage was set at 5 kV.

### 2.5. Calculation Methodology

According to the modified rule of mixtures [27,28], the tensile strength of a fiber-reinforced composite material (σ_t_^c^) as a function of the volume fraction of fibers (V^F^) is given by:

σ_t_^c^ = f_c_ × σ_t_^F^ × V^F^ + (1 − V^F^) × σ_t_^m*^ (1)

where σ_t_^m*^ is the tensile strength of the matrix at composite fracture, σ_t_^F^ is the intrinsic tensile strength of fibers, and f_c_ is a coupling factor. The product of the latter two parameters corresponds to the slope of σ_t_^c^ against V^F^. At the same time, the coupling factor can be expressed as the product of two contributions:

fc = χ_1_ × χ_2_ (2)

In Equation (2), χ_1_ accounts for the orientation of the fibers within the matrix, which depends mainly on processing. Regarding χ_2_, it is a function of the dimensions of the fibers and their compatibility with the matrix. Nonetheless, not all the fibers present in the composite have the same capacity for stress transfer. For the case of thermoplastic matrices reinforced with imperfectly aligned discontinuous fibers, we will consider the model of Kelly and Tyson with modifications proposed by Bowyer and Bader [29,30]:

(3) $$\sigma_{t}^{c}={\;\chi }_{1}\left[\sum_{i}\frac{{\tau \;l}_{i}^{F}{\;V}_{i}^{F}}{d^{F}}+\sum_{j}\left(\sigma_{t}^{F}{\;V}_{j}^{F}\;\left(1-\frac{\sigma_{t}^{F}{\;d}^{F}}{4{\;\tau \;l}_{j}^{F}}\right)\right)\right]+\left(1-V^{F}\right)\times {\;\sigma }_{t}^{m*}$$

where τ is the interfacial shear strength, l^F^ is the length of the recovered fibers, and d^F^ is their mean diameter. It may be noted, from Equations (1) and (2), that the brackets in the first term of Equation (3) correspond to σt^F^ × V^F^ × χ_2_. The subscript i refers to subcritical fibers, i.e., those whose length is lower than the critical length [27], meaning that they cannot withstand the maximum stress transferred from the matrix. The subscript j corresponds to supercritical fibers, which are capable of maximum stress transfer (at least in the middle of the fiber). Therefore, Equation (2) can also be expressed as:

Σ_t_^c^ = χ_1_ (X + Y) + Z (4)

These separate contributions can be used to calculate both τ and χ_1_ through an iterative procedure [30]. Two points are selected from stress–strain curves, namely, (ε_t,1_^c^,σ_t,1_^c^) and (ε_t,2_^c^,σ_t,2_^c^), where ε_t,1_^c^ = ε_t_^c^/3 and ε_t,1_^c^ = 2 × ε_t_^c^/3 Then, the value of τ that fulfills Equation (4) is used in Equation (2) to obtain χ_1_.

(5) $$\frac{X_{1}+Y_{1}}{X_{2}+Y_{2}}=\frac{\sigma_{t,1}^{c}-Z_{1}}{\sigma_{t,2}^{c}-Z_{2}}$$

As in previous works [26], the product χ_1_ × X will be referred to as X’ and the product χ_1_ × Y will be referred to as Y’. This way, *Z* is the contribution of the matrix to the strength of the composite, X’ is the contribution of subcritical fibers, and Y’ is the contribution of supercritical fibers.

## 3. Results

### 3.1. Effects on Chemical Composition and Polarity

Aiming to grasp an understanding of interfacial interactions, Table 1 displays the basic composition, structural properties, and dimensions of SBHFs, in comparison to those of hemp strands. In any case, SBHFs were found to contain mostly cellulose with a high degree of crystallinity (87%), while the original hemp strands were rich in inherently amorphous macromolecules.

In another context, pulped and bleached fibers displayed less MGCh adsorption capacity and subsequently less polarity. This is due to changes not only in surface morphology but also in the chemical composition. The hemicellulose fraction, which is larger in UHSs, contains a small but significant proportion of negatively charged glucuronic acid groups [31]. Furthermore, lignin, even without alkaline treatments, has a high density of electron-rich sites that were capable of interacting with the electron-acceptor groups of MGCh. In any case, the surface polarity of PLA was measured as 2.8 µeq MGCh/g, which is closer to the value of SBHFs than to the value of UHSs. In other words, pulping and bleaching helps decrease the high difference in polarity between the fibers and the matrix.

### 3.2. Features of Biocomposites

**Figure 2:**
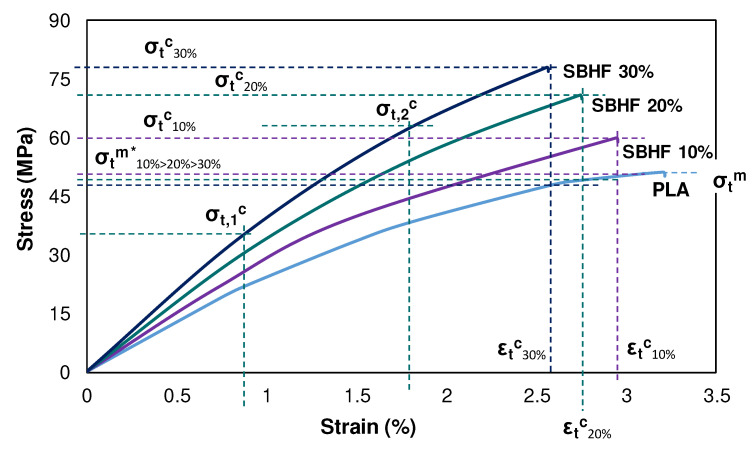
Mean stress–strain curves of PLA/SBHF composites, indicating the strength of the matrix at composite fracture in each case. Percentages are based on weight.

The tensile performance of PLA/SBHF composites is displayed in Figure 2 (stress-strain curves) and Table 2 (strength data). Stress–strain curves identify σ_t_^c^, σ_t_^m*^, and ε_t_^c*^. The σ_t,1_^c^ and σ_t,2_^c^ values that were chosen for Equation (5) are exemplified for the case of SBHF 30 wt.%. In comparison to the PLA matrix, the tensile strength of composites increased by up to 52% when the mass fraction of fibers was 30 wt.% (Table 2). This corresponded to a volume fraction (V^F^) of 0.262. In contrast, SBHF additions as high as 40 wt.% or higher severely hindered the melt flow and impaired the production of specimens.

It is consuetudinary to regard a linear trend of σ_t_^c^ with V^F^, as can be appreciated from Table 2 (Pearson’s r ~0.99), as an indicator of proper interfacial interactions between the matrix and the filler [26].

Moreover, as commonly found for fiber-reinforced thermoplastics [32], whereas the maximum stress that the composite can withstand increased, the strain it underwent before failure decreased. In other words, the biocomposite was, at the same time, stronger and less plastically deformable than PLA. As a result, and as shown in Table 2, PLA/SBHF (30 wt.%) composites are much more brittle than polypropylene (PP) reinforced with GF and a coupling agent, maleic anhydride grafted polypropylene (MAPP). The Discussion section deals with this comparison in more detail.

**Figure 3:**
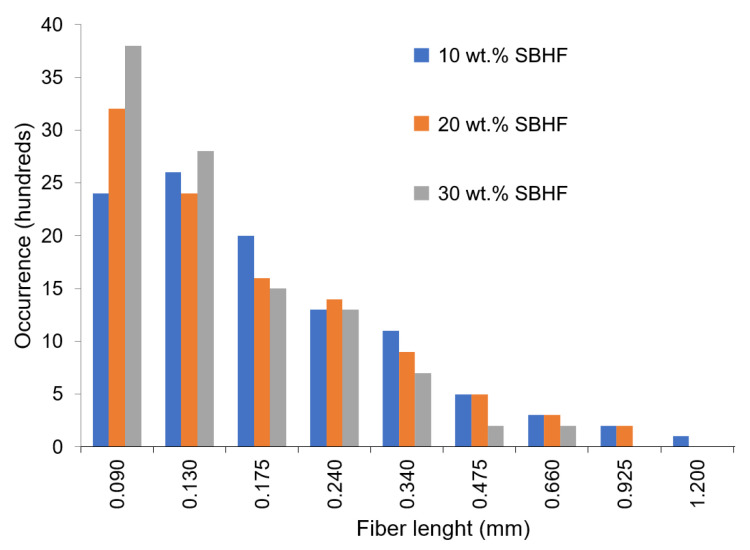
Length distribution of the fibers recovered from PLA/SBHF composites. The horizontal axis indicates the median of each length interval considered.

The histogram for the asymmetrical length distribution of recovered fibers is provided in Figure 3. While d^F^ did not decrease significantly, l^F^ consistently decreased with the proportion of SBHFs in the composite, and its distribution became skewed towards the short end (left in Figure 3), due to fibers being submitted to higher shear stress. Fiber fracture takes place during the whole process, including mixing at high temperatures, extruding twice, and injection molding [34]. Overall, the average length (weighted in length) was reduced by factors of 2.07, 2.30, and 2.47 when the fiber load was 10 wt.%, 20 wt.%, and 30 wt.%, respectively (Table 3).

**Figure 4:**
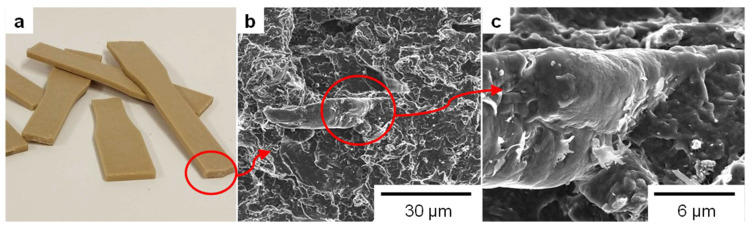
Pictures (**a**) and cross-sectional micrographs at two levels of magnification, 1000× (**b**) and 5000× (**c**), showing the fracture section of dog-bone specimens of PLA/SBHF biocomposites.

As can be observed from Figure 4a, different PLA/SBHF specimens displayed a macroscopically homogeneous and identical color at the fracture section. Color did not differ significantly from that of unreinforced PLA. The micrographs in Figure 4b,c correspond to a 20 wt.% fiber load. We can appreciate voids across the structure of the matrix and truncated fibers, resulting from the transfer of tensile stress from the former to the latter. In particular, Figure 4c highlights the fiber–matrix interphase, suggesting not only mechanical anchoring but also a well-bonded system.

### 3.3. Micromechanics of the Tensile Strength

Average fiber dimensions are presented in Table 3, along with key micromechanical parameters and the inputs to calculate them. Fiber orientation (χ_1_~0.3) resembles the values obtained from our previous experiments with the same machinery [26]. Indeed, during injection, only in the skin layer are fibers consistently oriented in the flux direction.

Regarding the mean intrinsic tensile strength of fibers (σ_t_^F^), the value displayed in Table 3 was computed through the Kelly and Tyson model, but it can be validated by other methods. As natural fibers tend to break by brittle fracture, undergoing little plastic deformation, σ_t_^F^ can be estimated as the product of the strain at failure of the composite and the tensile modulus of fibers. Bowyer and Bader used a similar approach to evaluate the contribution of the fibers at different strains [30]. Undoubtedly, this returns a value higher than the real intrinsic tensile strength of the fiber, because such fibers will break at lower strains than the composite. Nonetheless, the resulting value, 894.9 MPa, can be used to establish an upper bound as a criterion of validity. Additionally, Migneault et al. [35] have estimated the maximum value of σ_t_^F^ as 2 × τ × l^F^⁄d^F^. The formula, used with our experimental data, returned a value of 887 MPa, within the tolerance interval of Kelly and Tyson’s estimation. All considered the value reported in Table 3 is lower than those from the other methods, ensuring a more cautious evaluation of the transfer of stress from the matrix to the fibers.

The values obtained for the interfacial shear strength, 28.0–28.6 MPa, lie closer to those predicted by von Mises’ criterion (τ~σ_t_^m^/√3) than by Tresca’s criterion (τ~σ_t_^m^/2) [36].

## 4. Discussion

### 4.1. Insights from Micromechanics: Contribution of Fibers

**Figure 5:**
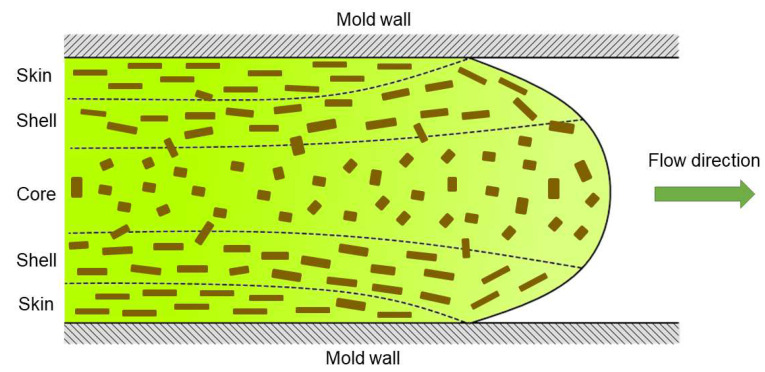
Orientation of reinforcement fibers across the matrix during injection molding.

According to Equation (3), if all fibers were perfectly aligned (χ_1_ = 1), the average tensile strength of the composite would be 259 MPa. While composites with highly aligned fibers can be produced by other methods [37], the injection molding process only attains flow-oriented fibers at the outer layers. At the core, fibers tend to become transversely oriented, as schematized in Figure 5 [38]. Hence, the orientation factor calculated (χ_1_~0.3) corresponds to an average value from different parts of the specimen.

The intrinsic tensile strength of SBHFs (roughly 850 MPa), even though falling short of that of GF, is significantly higher than that of lignocellulosic fibers [33]. This highlights the relevance of chemical (or semichemical) pulping and subsequent bleaching. On one hand, the removal of amorphous components leaves a highly crystalline network of cellulose-cellulose interactions, attaining higher σ_t_^F^. On the other, the surface of fibers is no longer prevented by attached lignin from H-bonding with PLA and among themselves. The latter is generally not desirable since it is detrimental to dispersion in the matrix. Indeed, it has been shown that coating natural fibers with a lignin layer enhances the dispersion of fibers in the matrix, regulating the polarity of the surface of fibers and leading to high gains in tensile strength [39]. However, that process implied additional stages and imparted, seemingly, certain loss in stiffness.

In any case, for PLA/SBHF, the resulting f_c_ is approximately 0.2 in all cases, which is indicative of proper fiber–matrix bonding in discontinuously reinforced composites [40]. It may be suggested that extensive mechanical processing, despite exposing fibers to high shear stresses, attained proper anchoring of them to PLA, overcoming to a certain extent the inherent dispersion issues of cellulose in thermoplastics.

**Figure 6:**
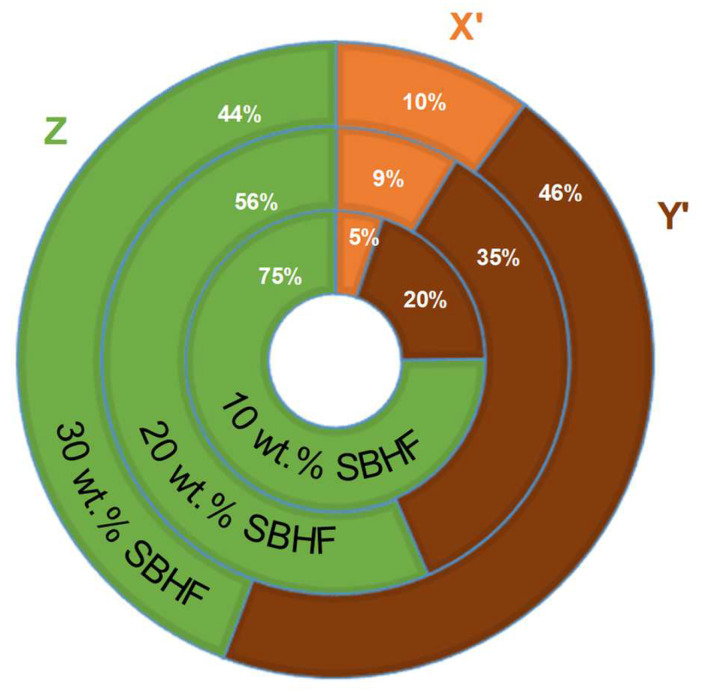
Proportional contributions of the matrix (Z), subcritical fibers (X’), and supercritical fibers (Y’) to the tensile strength of each PLA/SBHF composite material.

The different contributions of subcritical and supercritical fibers, along with the contribution of the matrix, are displayed in Figure 6. As expected, upon loading the composite with more SBHFs, their contribution to the tensile strength of the composite increased. In all cases, the ratio of the importance of subcritical fibers to the total contribution of fibers was roughly 1:4.

### 4.2. Postulating Fiber–Matrix Interactions

**Figure 7:**
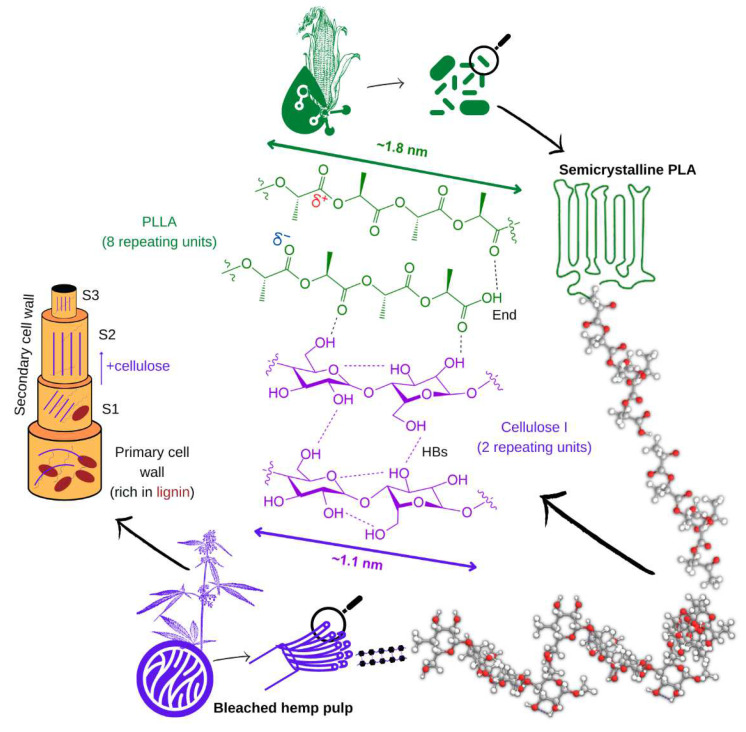
Structures of the repeating units of cellulose (crystalline polymorph I) and PLA (as intrinsically isotactic PLLA), highlighting their interactions and schematizing supramolecular structures.

PLA as the continuous phase, owing to their electronegative oxygen atoms, is more likely to establish strong interactions with cellulosic fibers (in terms of bond dissociation energy) than, e.g., PP. Once fibers are mechanically anchored in the matrix, the quality of the interphase depends, at the level of macromolecules, on a complex sum of intermolecular interactions at different planes. The most energetic ones are hydrogen bonds (HBs), in which the equatorial –OH groups of cellulose on the surface of reinforcement fibers work as donors (in the absence of water), whereas the electronegative oxygen atoms in PLA’s > C=O bonds act as acceptors. The latter groups are presented in-plane in Figure 7. It should be remarked that only a relatively small part of the cellulose chains, i.e., those at the surface of the fibers, can participate in HBs with the matrix.

In light of the high degree of crystallinity of SBHFs, cellulose chains in each of the dispersed fibers are mostly self-bonded. Figure 7 shows a segment of the parallel chains of cellulose I, spaced roughly 0.25 nm. It can be noted that, in general, natural fibers used as reinforcement for thermoplastics are mainly constituted by cellulose I, while cellulose II is known to enhance the mechanical properties of elastomers [41]. This network of cellulose–cellulose HBs is a known drawback of combining cellulose with PLA, since the former’s hydrophilic chains tend to associate with each other and with bound water, rather than with the thermoplastic matrix [6]. However, the strength of O–H…O=C–O HBs, as in the case of polysaccharide–polyester interactions, should not be overlooked. Ester groups only act as acceptors, but with bond energies above 10 kcal/mol [42].

In another context, even though HBs account for the most intense attractive interactions, the additive contribution of dipole–dipole and dispersive forces should be taken into account as well [43]. The polar character of all C–O and C=O bonds in PLA implies permanent dipoles. Furthermore, since every hydroxyl group of cellulose holds an equatorial position, the axial plane of each anhydroglucose unit is available for significant dispersive interactions (0.1–1 kcal/mol) with PLA’s methyl groups (out-of-plane in Figure 7).

Lengths of polymer segments in Figure 7 assume a rod-like conformation and C–C/C–O–C bond angles of approximately 109°. This way, there are approximately 5.5 HB donor groups per nm of cellulose and 2.2 HB acceptor groups per nm of PLA. However, only those at the surface of SBHFs are expected to H-bond with PLA, and this is why bleached short fibers are advantageous since they offer more surface area. All considered, Figure 3 depicts 1.1 intermolecular HBs per nm of interphase. Unlike cellulose, PLA is incapable of H-bonding with itself, except for the carboxyl group at the end of each chain.

### 4.3. Comparing PLA/SBHF to PLA/UHS, PLA/GF, and PP/GF

As schematized in the left part of Figure 7, the primary cell wall of UHSs, as in most plant fibers, is where most of the lignin is located. In the first layer of the secondary wall (S1), fibrils encompassing both cellulose and hemicellulose can be nearly perpendicular to the axis of the fiber. At least in the case of hemp, the S2 layer accounts for the largest part of the cross-sectional area, it is richer in cellulose (~80%), and fibrils are almost perfectly coaxial to the fiber [44]. Alkaline pulping especially attacks the primary wall, removing a large proportion of lignin and impacting the surface of the fibers. Bleaching is more selective towards lignin, whose complete elimination enhances both cellulose–PLA and cellulose–cellulose interactions. Moreover, the higher amorphous fraction in UHSs is tightly related to their lower intrinsic tensile strength in comparison to delignified pulps.

As of today, GF reinforcements are generally preferred over natural fibers, due to their better mechanical performance for most applications, including the higher intrinsic tensile strength of GF [10]. Due to its hydrophilic nature, it is usually sized (e.g., with silane coupling agents) or used along compatibilizers when combined with thermoplastic matrices. Reports on PLA/GF composites are very scarce, but those from RTP Company [13] attained tensile strength enhancements as high as 84%, from 62 to 114 MPa (Table 2). Nonetheless, as far as we are concerned, the manufacturer provided no clarifications regarding the manufacturing process. Likewise, although measurements from standard assays are meant to be intercomparable, sources of systematic error when considering samples from different laboratories should be taken into account.

PP is one of the most common thermoplastic materials for composites, GF being most often the dispersed phase [11,45]. As reported in a previous work of ours [33], PP/30 wt.% sized GF composites attain tensile strength values of 58.5 MPa, signifying an increase of 120% over the matrix (Table 2). With MAPP as a compatibilizer, σ_t_^c^ was as high as 79.9 MPa. Therefore, PLA/SBHF composites with the same fiber load (30 wt.%) significantly outperformed PP/sized GF in terms of tensile strength and approached the strength of PP/MAPP/GF. This is mainly due to the contribution of the matrix since the tensile strength of PLA (51.2 MPa) is higher than that of PP (27.6 MPa).

However, PLA is more brittle than PP, withstanding little plastic deformation. Hence, their range of applications is as broad as those of PP/GF materials (e.g., automotive, home appliances, construction) [46], excluding those for which the capability to withstand significant plastic deformation is required.

Another advantage of PP/GF composites is the possibility to further increase the fiber load, as PP/MAPP/40 wt.% GF materials may reach tensile strength values as high as 101 MPa [13]. In any case, the ability of PLA/30 wt.% SBHF to withstand tensile stress approaching or even surpassing that of PP/30 wt.% GF is noteworthy, and it does so with lower environmental impact [47]. Moreover, it should be stressed that SBHF with σ_t_^F^~850 MPa yielded enhancements of 52% on the tensile strength of the composite, while GF with σ_t_^F^~2500 MPa [33] yielded Δσ_t_^c^ = 84% on PLA [13]. Thence we can conclude that the reinforcement efficiency [48] of SBHF is higher than that of GF.

## 5. Conclusions

As the primary objective, this work explored composites with PLA and SBHFs as a greener alternative to conventional PP/GF materials. Overall, the biocomposite with a 30 wt.% SBHF reinforcement was shown to match or even surpass conventional PP/GF materials with the same fiber load, at least in terms of their tensile strength. Indeed, its average value increased from 51.2 to 77.8 MPa, although the natural fiber-reinforced composite withstood even less plastic deformation than unreinforced PLA. In comparison, the result for UHS-reinforced PLA was 55.7 MPa. Furthermore, tensile strength followed a linear trend with the volume fraction of natural fibers (r = 0.99), which indicates good interaction at the fiber–matrix interphase. The intensive mechanical blending process shortened fibers by factors of 2–2.5. At the surface of these fibers, which had less polarity and offered more bonding area than UHSs, the hydroxyl groups of cellulose may establish over 1.1 HBs with an adjacent PLA chain per nm of interphase.

A micromechanical analysis proved the importance of pulping and bleaching to obtain coupling factors around 0.2. The interfacial shear strength was over 28 MPa. Thence it can be concluded that the quality of the interphase was satisfactory. The removal of lignin is important not only to attain proper bonding between a discontinuously dispersed phase and a thermoplastic matrix, but also to grant intrinsic tensile strengths above 800 MPa.

## Figures and Tables

**Table 1:** Basic chemical composition and key properties of SBHFs, in comparison to the untreated strands.

	SBHF	UHS
Ash (wt.%)	0.31 ± 0.04	2.68 ± 0.67
Extractives (wt.%)	0.38 ± 0.05	3.2 ± 0.2
Total lignin (wt.%)	0.43 ± 0.8	5.12 ± 0.25
Cellulose (wt.%)	91.3 ± 0.6	73.2 ± 1.9
Hemicellulose (wt.%)	7.9 ± 0.5	11.3 ± 1.2
Average dimensions	Short fibers, 730 µm × 21 µm	Long bundles, ~200 µm–wide
Content of fines (% in length)	47	--
Crystallinity index (%)	87.0	78.5
Surface polarity (µeq MGCh/g)	12.8	28.7

**Table 2:** Tensile strength and relative enhancements over the PLA matrix that were attained by PLA/SBHF, in comparison to PLA/untreated hemp, PLA/GF, and PP/GF. The elongation and the volume fraction are also reported.

Material	Reinforcement	V^F^	σ_t_^c^ (MPa)	Δσ_t_^c^ (%)	ε_t_^c^ (%)	Reference
PLA	0 wt.%	0	51.2 ± 0.1	--	3.2 ± 0.1	This work
PLA/SBHF	10 wt.%	0.085	59.9 ± 0.2	17.0	2.9 ± 0.1	This work
	20 wt.%	0.171	70.7 ± 0.4	38.1	2.7 ± 0.2	
	30 wt.%	0.262	77.8 ± 0.8	52.0	2.6 ± 0.2	
PLA/hemp strands	30 wt.%	0.264	55.7 ± 0.2	8.8	2.0	This work
PLA/GF ^1^	30 wt.%	0.175	114	83.9	N/A	[13]
PP/sized GF	30 wt.%	0.136	58.5	120	3.0	[33]
PP/MAPP/GF	30 wt.%	0.136	79.9	189	4.4	[33]

^1^ GF treatment unspecified in the source. Enhancement (Δσ_t_^c^) refers to their non-reinforced PLA (62 MPa).

**Table 3:** Assessment of tensile strength at the level of constituent materials: inputs and results from the Bowyer and Bader solution (Equation (3)) and the modified rule of mixtures (Equation (1)).

SBHF Proportion	10 wt.%	20 wt.%	30 wt.%
V^F^	0.085	0.171	0.262
σ_t_^m*^ (MPa)	49.2	48.1	46.7
d^F^ (µm)	20.5	20.5	20.4
l^F^ (µm)	353	318	295
χ_1_	0.306	0.300	0.297
τ (MPa)	28.6	28.1	28.0
Mean σ_t_^F^ (MPa)	850 ± 49		
χ_2_	0.67	0.71	0.65
Mean σ_t_^F^ (MPa)	850 ± 49		
Mean f_c_	0.20 ± 0.1		

## Data Availability

All data explicit in the manuscript or else available at request.
